# Multi-contrast machine learning improves schistosomiasis diagnostic performance

**DOI:** 10.1371/journal.pntd.0012879

**Published:** 2025-08-04

**Authors:** María Díaz de León Derby, Charles B. Delahunt, Ethan Spencer, Jean T. Coulibaly, Kigbafori D. Silué, Isaac I. Bogoch, Anne-Laure Le Ny, Daniel A. Fletcher

**Affiliations:** 1 Department of Bioengineering, University of California, Berkeley, Berkeley, California, United States of America; 2 Global Health Labs, Inc, Bellevue, Washington, United States of America; 3 UFR Biosciences, Université Félix Houphouët-Boigny, Abidjan, Côte d’Ivoire; 4 Centre Suisse de Recherches Scientifiques en Côte d’Ivoire, Abidjan, Côte d’Ivoire; 5 Division of General Internal Medicine, Toronto General Hospital, University Health Network, Toronto, Canada; 6 Division of Infectious Diseases, Toronto General Hospital, University Health Network, Toronto, Canada; 7 Department of Medicine, University of Toronto, Toronto, Canada; 8 Biological Systems and Engineering Division, Lawrence Berkeley National Laboratory, Berkeley, California, United States of America; 9 Chan Zuckerberg Biohub, San Francisco, California, United States of America; 10 California Institute for Quantitative Biosciences (QB3), University of California, Berkeley, Berkeley, California, United States of America; Khon Kaen University Faculty of Medicine, THAILAND

## Abstract

Schistosomiasis currently affects over 250 million people and remains a public health burden despite ongoing global control efforts. Conventional microscopy is a practical tool for diagnosis and screening of *Schistosoma haematobium*, but identification of eggs requires a skilled microscopist. Here we present a machine learning (ML)-based strategy for automated detection of *S. haematobium* that combines two imaging contrasts, brightfield (BF) and darkfield (DF), to improve diagnostic performance. We collected BF and DF images of urine samples, many of them containing *S. haematobium* eggs, during two different field studies in Côte d’Ivoire using a mobile phone-based microscope, the SchistoScope. We then trained separate egg-detection ML models and compared the patient-level performance of BF and DF models alone to combinations of BF and DF models, using annotations from trained microscopists as the gold standard. We found that models trained on DF images, and almost all BF and DF combinations, performed significantly better than models trained on BF images only. When models were trained on images from the first field study (n = 349 patients, 748 images of each contrast), patient-level classification performance on patient images from the second study (n = 375 patients, 752 images of each contrast) met the WHO Diagnostic Target Product Profile (TPP) sensitivity and specificity for the monitoring and evaluation use case (sensitivity for all models and combinations was >75% when evaluated at a confidence score threshold that resulted in specificity >96.5%). When we used images from both field studies for the training set, performance of the models was improved. Overall, this work shows that the use of DF and BF increases the performance of ML models on images from devices with low-cost optics, while retaining the portability, power, and time-to-results of the WHO’s diagnostic TPP. DF requires no additional sample preparation and does not increase the complexity of the imaging system. It thus offers a practical means to improve performance of automated diagnostics for *S. haematobium* as well as other microscopy-based diagnostics.

## Author summary

Schistosomiasis is a neglected tropical disease that impacts hundreds of millions of people worldwide. Patients with *Schistosoma haematobium* shed parasite eggs in their urine, which can be used as a diagnostic marker of disease. However, identification of those eggs in patient samples normally requires a microscope and trained microscopist. In this work, we show that machine learning models trained on two imaging contrasts, brightfield and darkfield, can improve performance of automated schistosomiasis diagnosis. Using a mobile phone-based microscope (the SchistoScope), we captured brightfield and darkfield images of patient samples during two visits to Côte d’Ivoire and then trained models to detect eggs in the images. When training on images from one visit (n=349 patients) and testing on images from the other visit (n=375 patients), we found that the darkfield model and combinations of brightfield and darkfield outputs improved the diagnostic performance compared to brightfield alone. When evaluated at a threshold that led to a specificity of 96.5%, sensitivity for the brightfield models was 76%, while sensitivity for darkfield and for combined models was ≥81%, meeting the WHO’s target metrics for monitoring and evaluation of schistosomiasis control programs. This use of multi-contrast machine learning with a mobile microscope has the potential to improve diagnostic testing for schistosomiasis and could be extended to other neglected tropical diseases.

## Introduction

Schistosomiasis is a neglected tropical disease (NTD) caused by parasitic flatworms that affects more than 250 million people worldwide, with an estimated 800 million people at risk of contracting the disease [1,2]. *Schistosoma haematobium* is one of the main species responsible for the disease’s morbidity and mortality. The lack of rapid, portable, and accurate diagnostic tools hinders infection control and elimination efforts in endemic regions.

The standard diagnostic strategy for *S. haematobium* is detection of parasite eggs in urine samples. This method typically involves urine filtration or centrifugation, followed by examination of the sample by a trained expert using light microscopy. These methods are time-consuming and require infrastructure and personnel that are often not available in resource-limited endemic regions. The World Health Organization (WHO) has identified the need for novel diagnostic tools that enable monitoring and evaluation of schistosomiasis control programs through their Diagnostic Target Product Profiles (TPP) [3]. Ideally, these tools should be portable, use battery-powered equipment, require minimal training for field workers, and have a time to result <2 hours.

One strategy to facilitate diagnosis of *S. haematobium* and other helminths at the point-of-care is to use portable platforms to image and automatically analyze patient samples. Several groups have developed novel imaging systems that, in combination with machine learning (ML) for image analysis, can be used to identify parasite eggs from urine and stool samples acquired in field settings [4–13]. These devices are versatile and pay close attention to robustness and user needs. ML algorithms for patient-level schistosomiasis diagnosis have shown a range of success from 83-96.3% sensitivity and 77-99% specificity, approaching or exceeding the WHO target performance [5,6,8,14]. However, these devices and algorithms are still not widely available and have not been fully validated for field use [4,15]. Some devices have long imaging and sample processing times (25-90 minutes) [7,9], and some require the samples to be transported to local laboratories for analysis [6,8,12], making them challenging to use for point-of-care detection and mapping of schistosomiasis in remote locations. Most imaging devices developed for egg detection are relatively heavy (>4 kg), use standard objective lenses, or require the use of a computer. Other portable approaches, including lens-less imaging [16,17], mobile phone-based microscopes [18–20], and 3D printed smartphone-based adapters for standard microscopes [21], have been developed for other diagnostic applications. Portable, low-cost microscopy with low-resolution imaging is a more workable but under-explored solution for field diagnosis of *S. haematobium*.

In this work, we use a low-cost, mobile phone-based microscope called the SchistoScope [22] and show how images taken with two contrasts—darkfield (DF), in addition to the standard brightfield (BF)—can be used to improve automated diagnostic performance for schistosomiasis. We previously demonstrated that the SchistoScope, a highly portable device (<1kg) that runs independent of mains power, can be used to simplify *S. haematobium* sample preparation and image acquisition, enabling collection of BF and DF images of patient samples in under 5 minutes. The SchistoScope performs well when compared to conventional on-site light microscopy, as shown in field studies in Ghana and Côte d’Ivoire [22–24], but lack of automated patient-level diagnosis with high sensitivity and specificity has been a limitation. Here, we use images acquired on the SchistoScope to train ML object detection models by fine-tuning an off-the-shelf YOLOv8 architecture [25] for *S. haematobium* egg detection.

### Contributions.

1. We show that training ML models on DF images improves the performance for *Schistosoma* egg detection, compared to models trained on BF images alone.
2. We demonstrate an automated diagnostic strategy that utilizes DF imaging to enable a device with low-cost optics to meet WHO requirements for the monitoring and evaluation of schistosomiasis control programs, including sensitivity and specificity, portability, no mains power, and time-to-result.
3. We collect and annotate a dataset of BF and DF images of *S. haematobium* that can be used for further development of machine learning models for egg detection.

## Materials and methods

### Ethics statement

This work contains patient data from two separate studies conducted in Côte d’Ivoire. The first study was conducted in March 2020 in the Azaguié region of Côte d’Ivoire [23]. Ethical permission for this study was granted by the Centre Suisse de Recherches Scientifiques en Côte d’Ivoire, Abidjan, Côte d’Ivoire (#054-19) and the University Health Network, Toronto, Canada (REB #14-8128). Permission was granted by the local Health District officer. School-age children between 5 and 14 years were invited to participate, and both signed parental consent and the children’s assent were required for inclusion.

The second study was conducted in November 2021, in the Koubi village near the Tiébissou district in Côte d’Ivoire [24]. Ethical permission for this study was granted by the local Health District officer, from the Comité National d’Éthique des Sciences de la Vie et de la Santé, Abidjan, Côte d’Ivoire (REB #186-21) and the University Health Network, Toronto, Canada (REB #21-5582). Community members over 5 years old were asked to participate. Adults provided written consent, and children were included if they assented and had written consent from a parent or guardian.

### Sample processing and image acquisition

**Fig 1:**
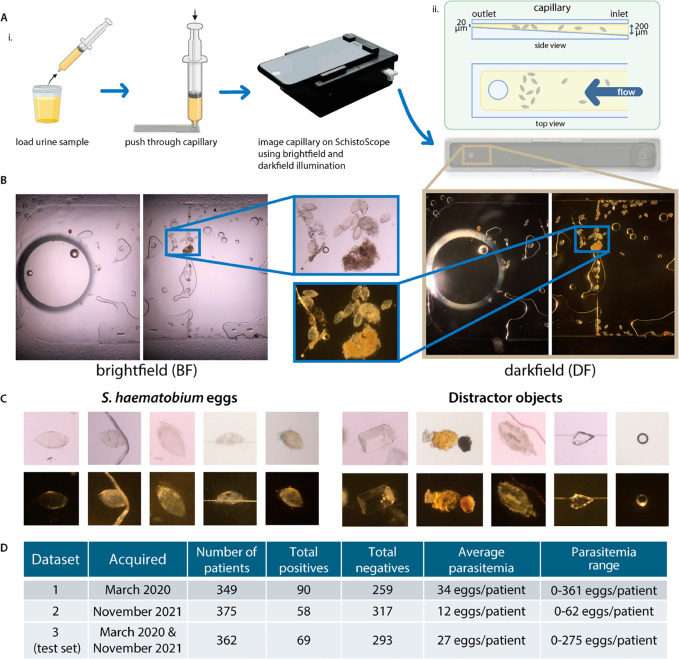
Sample processing, image acquisition and dataset information. **A**: Diagram showing urine sample processing using a capillary and image acquisition with SchistoScope (Ai). Diagram showing capillary dimensions and egg trapping (Aii). Partially created with BioRender.com **B**: Example images in BF and DF of two fields of view of a capillary containing *S. haematobium eggs* and other debris. **C**: Examples of *S. haematobium* eggs and distractor objects trapped in capillaries and imaged with the SchistoScope. **D**: Information about Datasets 1, 2, and 3. Dataset 1 was collected in March 2020 [23] and Dataset 2 was collected in November 2021 [24], in different field sites in Côte d’Ivoire. Dataset 3 is a combination of Datasets 1 and 2 and was randomly split into train and test sets—the table shows information for the test set of Dataset 3. In the field studies where they were collected, the percentage of urine specimens examined that were found to contain *S. haematobium* eggs using conventional light microscopy was 20.6% for Dataset 1 and 13.4% for Dataset 2.

Sample processing and image acquisition with the SchistoScope are illustrated in Fig 1A and described in more detail in [22]. For each patient, urine samples were collected in plastic cups and loaded into a 10mL syringe (Fig 1Ai). The syringe was connected to a custom injection-molded disposable plastic capillary designed to trap *S. haematobium* eggs. The capillary had a rectangular cross-section that tapered down from a height of 200μm at the inlet to 20μm near the outlet hole, trapping and concentrating eggs and other debris as the urine flowed into the capillary and exited through the outlet (Fig 1Aii). After filtration, the capillary was inserted into the SchistoScope and image acquisition began. For this, the capillary was translated in one axis, and images of six distinct fields of view (FOV) were acquired. The SchistoScope was designed such that approximately the same capillary locations were imaged for each patient sample, making FOVs consistent across patients. Most *S. haematobium* eggs were consistently trapped in the two FOVs closest to the outlet hole, which were the first to be acquired in the imaging routine. In some high parasitemia cases, additional eggs were found in FOVs closer to the capillary inlet, and more FOVs along the capillary could be imaged or analyzed for higher egg counting accuracy. Based on previous field studies, we do not expect all eggs found in a urine sample to be captured by the capillaries [22].

Each FOV was imaged using both BF and DF illumination. We implemented BF imaging with an LED illuminator below the sample and DF imaging with an LED illuminator above the sample, oriented at an angle such that unscattered light was not collected by the camera lens. To capture BF and DF images of a single FOV, we turned on the BF illuminator, focused automatically, captured an image, turned off the BF illuminator, turned on the DF illuminator, refocused, captured an image, and then turned off the DF illuminator. The entire imaging sequence, including autofocus, took an average of 60 seconds (range 47-72 sec).

The SchistoScope images are 4032 x 3024 pixels, with pixel pitch ≈1 μm/pixel. The optical resolution of the SchistoScope is estimated to be <5 μm [22]. Example images of two distinct FOVs for one capillary, captured in BF and DF, are shown in Fig 1B. Due to the tapered design of the capillaries, most eggs were trapped in the region near the outlet hole, corresponding to the first two imaged FOVs in each capillary. Examples of *S. haematobium* eggs and “distractor objects”, non-egg debris in urine samples, that were trapped in capillaries and imaged are found in Fig 1C.

### Dataset preparation

The images used for this work were acquired during two separate visits to *S. haematobium*-endemic regions in Côte d’Ivoire. We created two datasets using the data from these two visits, described below. These datasets have been fully described and are publicly available [26]. Information for these datasets is summarized in Fig 1D.

The first field visit was completed in the Azaguié region in March of 2020, as described in [23]. One hundred and seventy individuals provided urine specimens, out of which 35 (20.6%) were found to contain *S. haematobium* eggs using standard light microscopy. Only three urine specimens contained more than 50 eggs per 10 mL of urine, meeting the WHO criteria for a high-burden infection [27]. From these urine specimens, 349 individual samples (consisting of 10mL of urine) were processed using capillaries and imaged using the SchistoScope, as described above. Given that these samples were processed using different volume fractions of urine, we will henceforth consider each individual sample to be a separate “patient” for the purposes of this work. We will refer to the images from this first field visit as “Dataset 1”. The average parasitemia (number of eggs per positive patient) in this dataset was 34 eggs/patient, as shown in Fig 1D. The parasitemia range was 0-361 eggs/patient. Most eggs were found in the two FOVs closest to the capillary outlet hole for each patient sample, i.e. these FOVs effectively contained the relevant contents of the 10 mL urine sample. Therefore, we used only these two images for the purpose of evaluating this dataset. We also included, for training purposes only, any additional FOVs that contained eggs (which only happened in 34 samples, all of which had very high parasitemia, and contained the most eggs in the first two FOVs). This resulted in a dataset of 748 total images for each contrast (BF and DF). Of those images, 186 BF images and 188 DF images were subsequently annotated as containing *S. haematobium* eggs.

The second field visit occurred in the Koubi village near the Tiébissou district in November of 2021, as described in [24]. Of the 365 urine specimens evaluated, 49 (13.4%) were positive for *S. haematobium* via standard light microscopy, with only 4 samples quantified as being a heavy burden infection with 50 or more eggs per 10mL of urine. From these urine specimens, 375 individual samples (consisting of 10mL of urine) were processed using capillaries and imaged on the SchistoScope. As above, we considered each individual sample a patient for the purposes of this work. We refer to the images from this field visit as “Dataset 2”. The average parasitemia in Dataset 2 was 12 eggs/patient and the parasitemia range was 0-62 eggs/patient, both lower than those of Dataset 1. The position of the capillaries in the SchistoScope was slightly shifted in this Dataset compared to Dataset 1, meaning that the second and third FOVs contained most *S. haematobium* eggs. We therefore included the images from those FOVs for all patients, resulting in 750 images per illumination contrast. Of those images, 92 BF images and 91 DF images were subsequently annotated as containing *S. haematobium* eggs.

We created “Dataset 3” by combining the images from both datasets into one, in order to assess whether our ML models were affected by distribution shifts, an effect commonly observed when using ML for medical tasks, where even small deviations from training conditions can lead to changes in performance [28–31]. We randomly split Dataset 3 into a train set and a test set. The average parasitemia for the test set of Dataset 3 was 27 eggs/patient and the parasitemia range was 0-275 eggs/patient.

### Image annotation

Patient sample images were annotated for the presence of *S. haematobium* eggs by a microscopist with experience in egg identification. These annotations were then verified by another microscopist. In cases of disagreement, a third microscopist was consulted. To carry out the annotations, each image was opened in Microsoft Paint, and the center of each visible egg was labelled with a blue dot. Objects that the annotator was unsure of and needed consultation with the second annotator were marked with a red dot. Unlabelled objects in the images were considered distractor objects, some of which are shown in Fig 1C.

### ML model training

**Fig 2:**
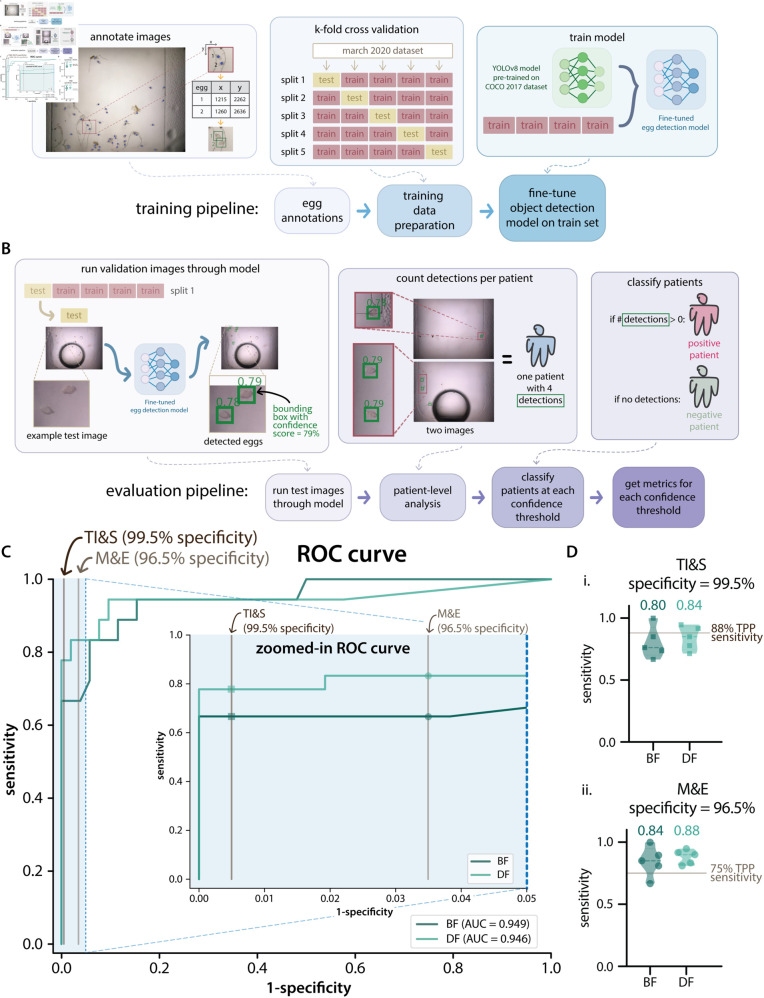
Dataset preparation, ML model training and evaluation of Dataset 1 k-fold splits. **A**: Diagram of the ML model training pipeline. First, *S. haematobium* eggs are annotated in dataset images. For Dataset 1, the patients are then split into 5 folds containing different subsets of training and test data. Transfer learning is done by fine-tuning the ML models (YOLOv8 pre-trained on the COCO 2017 dataset) using the training set for each split. **B**: Diagram of the model evaluation pipeline. After training, the test images are run through the trained model, generating bounding boxes surrounding detections with a confidence score assigned by the model. The number of detections above a certain confidence score threshold are counted for each patient. Each patient is represented by the first two images of a capillary. Subsequently, patients are classified as positive or negative depending on the presence or absence of detections with a confidence score above a given threshold. Sensitivity and specificity metrics are calculated on a patient population level. **C**: Full and zoomed-in receiver operator characteristic (ROC) curves for the first split of the data of Dataset 1, showing results for the BF and DF ML models and the area under each curve. The partial ROC curve is displayed as an inset of the full curve, it shows specificity values from 95% to 100%. The vertical lines indicate the targeted specificity for the transmission interruption and surveillance (TI&S) and monitoring and evaluation (M&E) TPP use cases (99.5% and 96.5%, respectively). **D**: Violin plots showing the patient-level sensitivity values for the 5 splits of Dataset 1 for the TI&S (Di) and M&E (Dii) use cases. The mean sensitivity is displayed above each violin and the targeted sensitivity for each use case is shown as a vertical line. Di shows the sensitivity at a threshold that resulted in 99.5% specificity. Dii shows the sensitivity at a threshold that resulted in 96.5% specificity. BF is brightfield and DF is darkfield.

Due to the relatively small size of our dataset, we used transfer learning to fine-tune pre-trained models to detect *Schistosoma* eggs (Fig 2A). We chose YOLOv8, developed by Ultralytics and pre-trained on the COCO 2017 dataset, in part because it can be exported to formats such as ONNX and TensorFlow Lite for use on mobile devices [25]. To fit the YOLOv8 input image size of 640 × 640 pixels, we cropped our 4032 × 3042 pixel images into 30 individual, partially overlapping, image tiles. We trained the YOLOv8 model using the “detect” task and the following training parameters: stochastic gradient descent optimizer, learning rate of 0.01, and batch size of 16.

Having data from two different field studies allowed us to use one as a holdout set to evaluate the performance of our trained ML models when tested on unseen data. In this work, we set aside Dataset 2 (since we had collected it second) and used Dataset 1 to explore ML model architectures and different ways to combine BF and DF images, as well as for ML hyperparameter tuning. We eventually used all of Dataset 1 to train a final pair of ML models, one for BF and one for DF images. We then used Dataset 2 as our holdout set, using the data to evaluate the models trained on Dataset 1.

We used 5-fold cross-validation (a standard technique to assess model stability), stratified by patient, during our exploratory model training phase using Dataset 1. We divided the 748 dataset images into five different “splits”, each containing a partially overlapping set of images for training, but a completely different set of images for testing. For each of these splits, we trained ML models on the train set images and then evaluated these models on the test set images, as illustrated in Fig 2A. To ensure that images from the same patient were not split between the train and test sets, images that originated from the same patient sample were assigned to the same “group” during k-fold cross-validation.

To ensure an even distribution of eggs and distractor objects across the splits, we divided the patients into 8 classes: classes 1-3 were positive patients with images that contained eggs in increasing amounts, classes 4-8 were negative patients that contained distractor objects in increasing amounts. We then used the ‘StratifiedGroupKFold’ function from the scikit-learn Python library [32], which splits the data into folds and assigns to each fold roughly equal proportions of each class and also stratifies by patient (i.e. all of a patient’s images are assigned to one fold).

When training the 5-fold split models using Dataset 1, we trained for 200 epochs. When training the final models using all of Dataset 1 to test on Dataset 2, we trained for 300 epochs. In all training instances, we trained separate object-detection models for the BF and DF images.

To compare our ML results with YOLOv8 to other commonly used models, we trained models using ResNet50 [33] and YOLOv5 [34] architectures. These models were trained and tested using the same 5-fold split of Dataset 1. ResNet50 models (pre-trained on the COCO2017 dataset) were trained using an Adam optimizer, a learning rate of 0.00167, and batch size of 29. YOLOv5 models (pre-trained on the COCO2017 dataset) were trained using the “detect” task and the following parameters: stochastic gradient descent optimizer, learning rate of 0.01, and batch size of 16.

### Patient classification

Our trained egg-detection models produce a series of detections in each test image that the model identifies as eggs, with an associated confidence score that goes from 1-100%. These detections are indicated by bounding boxes (Fig 2B). Since patient-level, not object-level, performance is what matters clinically, we converted the object-level detections to patient-level diagnostic classification as follows:

First, after running each individual image (composed of 30 image tiles) through the trained model, we combined the detections from the two images corresponding to each patient (Fig 2B). We then evaluated whether each patient would have been classified as positive or negative as we varied a threshold on the confidence score. A patient was considered positive if there was at least one detected object with a confidence score greater or equal to the threshold in any of the images for a patient. Otherwise, the patient was negative. This method applies the patient diagnosis framework in [28], where the noise floor is set to 0 due to the high accuracy of the detection algorithms used. The object-level precision-recall curves for all splits of the BF and DF models trained and tested on Dataset 1 are shown in S1 Fig. All other results shown in this work are at the patient-level.

### Evaluation metrics

We evaluated our ML models at the patient-level in the test dataset by calculating sensitivity and specificity, using the presence of eggs that were captured in the capillary, imaged by the SchistoScope, and annotated by an expert as the ground truth. We then compared the results to the target metrics for each schistosomiasis diagnostic use case in the WHO Diagnostic Target Product Profiles (TPP) for schistosomiasis control programmes [3].

The following equations define *sensitivity* (Eq 1) and *specificity* (Eq 2):

$$\text{Sensitivity}=\frac{\text{True Positives}}{\text{True Positives + False Negatives}}$$ (1)

$$\text{Specificity}=\frac{\text{True Negatives}}{\text{True Negatives + False Positives}}$$ (2)

Consistent with convention, True Positive patients are those that were annotated as having *S. haematobium* eggs and were classified as positive by the ML model, while False Negatives are patients that were annotated as having eggs but were classified as negative by the ML model. True Negative patients were both annotated and classified as negative, and False Positives were annotated as negative by human annotators but classified as positive by the ML model.

To show how patient-level sensitivity and specificity depend on the threshold confidence score for model detections, we generated receiver operator characteristic (ROC) curves for each model (BF and DF), which plot sensitivity, or True Positive Rate (TPR), vs 1-specificity, or False Positive Rate (FPR).

To assess the performance of our ML models in the context of schistosomiasis diagnostics, we evaluated whether we would meet the target metrics established in the WHO TPP. The TPPs are used to guide the development of new diagnostic tools for schistosomiasis for two use cases: (i) Monitoring and Evaluation (M&E) and (ii) Transmission Interruption and Surveillance (TI&S). The TPPs outline the target characteristics of a suitable diagnostic test in categories such as portability, training requirements, throughput, time to results, and clinical sensitivity and specificity. The target sensitivity and specificity for both use cases are shown in Table 1.

**Table 1:** Diagnostic Target Product Profile (TPP) requirements.

	TI&S	M&E
**sensitivity**	88%	75%
**specificity**	99.5%	96.5%

WHO TPP requirements for Monitoring and Evaluation (M&E) and Transmission Interruption and Surveillance (TI&S) of schistosomiasis control programmes.

We focused our performance analysis on the relevant regions of the ROC curve where specificity was above what is targeted by each WHO use case. Fig 2C shows the full ROC curves for one of the splits (split 1) of the BF and DF ML models trained and evaluated on subsets of Dataset 1, together with a zoomed-in portion of the ROC curve showing the specificity values above 95%. The two vertical lines indicate the specificity values targeted by both of the TPP use cases (96.5% for M&E and 99.5% for TI&S).

To directly compare performance with the TPP use cases, we took the sensitivity at the confidence threshold that resulted in the patient-level specificity targeted by each use case. That is, we set the operating point by requiring that the model meet the specificity in the TPP, then assessed whether it also met the TPP’s sensitivity [28]. Fig 2D shows the sensitivity values for each of the splits of Dataset 1 when evaluated at the targeted specificity for the TI&S (top) and M&E (bottom) use cases. The targeted sensitivity values for each use case are displayed as a horizontal line.

### Multi-contrast combinations

**Fig 3:**
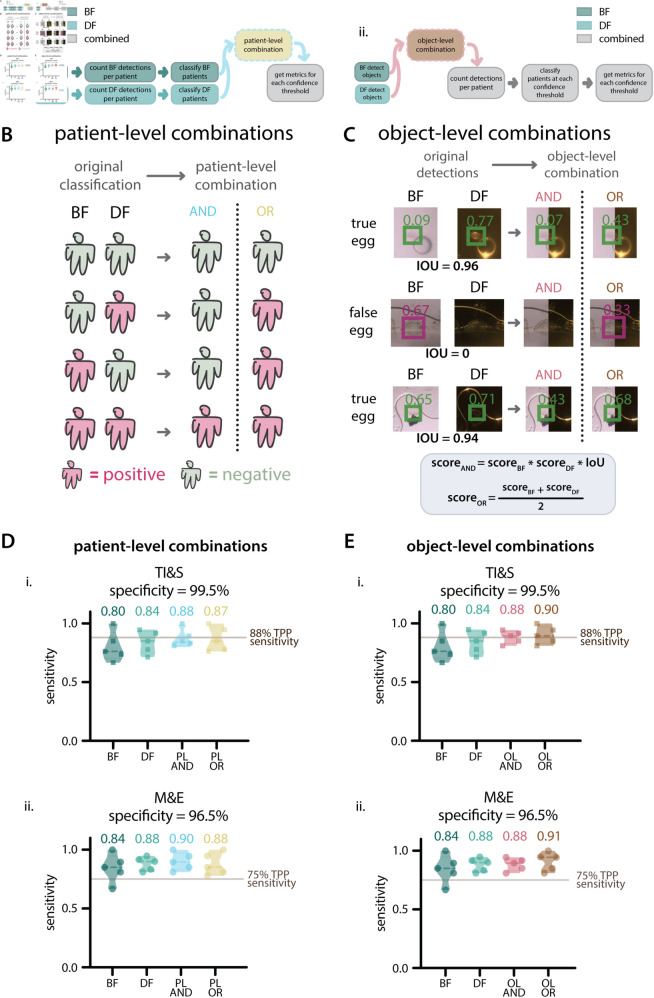
Contrast combination rubrics and patient-level sensitivity on 5-fold splits, Dataset 1. **A**: Combination pipelines. Ai: Diagram of the patient-level combination pipeline. Aii: Diagram of the object-level combination pipeline. **B**: Truth table for patient-level combinations, showing the four possible combinations of patient classifications based on BF and DF models individually, followed by the result after patient-level combinations. Positive patients are shown in magenta and negative patients are shown in green. **C**: Examples of object-level combinations on three objects in the images, showing original confidence scores assigned by BF and DF models, followed by resulting confidence scores after each combination. Green boxes represent true positive detections and magenta boxes represent false positive detections. **D**: Violin plots showing the sensitivity values after applying patient-level combinations to the 5 splits of Dataset 1 for the TI&S (Di) and M&E (Dii) use cases. The mean sensitivity is displayed above each violin and the targeted sensitivity for each use case is shown as a vertical line. Di shows the sensitivity at a threshold that resulted in 99.5% specificity. Dii shows the sensitivity at a threshold that resulted in 96.5% specificity. ‘BF’ is brightfield, ‘DF’ is darkfield, ‘PL AND’ is patient-level AND, ‘PL OR’ is patient-level OR, ‘OL AND’ is object-level AND, ‘OL OR’ is object-level OR. **E**: Violin plots showing the sensitivity values after applying object-level combinations to the 5 splits of Dataset 1 for the TI&S (Ei) and M&E (Eii) use cases. The mean sensitivity is displayed above each violin and the targeted sensitivity for each use case is shown as a vertical line. Ei: sensitivity at a threshold that resulted in 99.5% specificity. Eii: sensitivity at a threshold that resulted in 96.5% specificity.

We explored different approaches to combine BF and DF images and assessed whether they would result in improved sensitivity and specificity. The pre-trained YOLOv8 models that we used for transfer learning use 3-channel images as an input. We thus trained separate 3-channel models for BF and DF images and then combined the model outputs with boolean AND or OR, at either object-level or patient-level, for a total of four combination methods. The workflow for these combinations is illustrated in Fig 3A. Converting BF and DF images into greyscale and merging them into a 3-channel image to train ML models (e.g., [BF(greyscale), BF(greyscale), DF(greyscale)]) did not yield good results.

For patient-level combinations, we first used the BF and DF model outputs to classify the patients as positive or negative separately for each contrast. After this, we used patient-level AND/OR operations to combine the BF and DF results and arrive at a final diagnosis. For patient-level AND, we called a patient positive only when both BF and DF classified them as positive. For patient-level OR, a positive classification for either BF or DF resulted in a positive combined classification (Fig 3B). After these combinations, we calculated the sensitivity and specificity for the test patient populations and generated ROC curves for the AND and OR cases.

For object-level combinations, we follow the same procedure as above by first separately evaluating images with the BF and DF models, which generates a list of bounding box detections for each contrast. We then apply AND/OR operations at the object level to generate new object scores, as described below, followed by patient-level classification (Fig 3Aii).

To generate new object scores from the BF and DF detections and scores, we:

(i) pair up each individual detection on a BF image with each individual detection on the DF version of that image. Each of these pairs consists of the xy coordinates for the bounding box detection in BF and in DF, as well as their associated confidence scores (${\text{score}}_{BF}$ and ${\text{score}}_{DF}$).
(ii) use the BF and DF xy box coordinates to calculate the intersection over union (IoU) for each detection pair. IoU goes from 0-1 and it measures the overlap between the bounding boxes. If the boxes overlap completely, the IoU is 1. If they are partially overlapping, the IoU is smaller. If the boxes are not overlapping, meaning that a particular object was only detected in one of the contrasts, the IoU is 0.
(iii) carry out object-level AND/OR operations to assign a new object score.
  (a) For AND, the score is given by:
    $${\text{score}}_{AND}={\text{score}}_{BF}\;*\;{\text{score}}_{DF}\;*\;IoU$$
    Because the IoU is zero for non-overlapping detections, the object-level AND score eliminates detections that are not represented in both BF and DF. This is a stringent filter, only detections where BF and DF agree on the presence of an egg make it through.
  (b) for OR, objects that are only found in BF or DF are not eliminated, but their confidence scores are reduced. To do this, we first eliminate all object pairs that are not overlapping (i.e. pairs with IoU of zero). We then go through the original detection lists for BF and DF, find any detections that are not represented in the combined list, and add them back to the list as “lonely detection pairs”. For these pairs, we assign a confidence score of zero to the missing contrast. For example, if an object is detected only in BF with score = ${\text{score}}_{BF}$, a lonely detection pair is added to the list with ${\text{score}}_{BF}={\text{score}}_{BF}$ and ${\text{score}}_{DF}=0$.
    After adding the lonely detections, we calculate the object-level OR score as:
    $${\text{score}}_{OR}=\frac{{\text{score}}_{BF}+{\text{score}}_{DF}}{2}$$
    When using the object-level OR combination, we are not removing objects that are only detected in either BF or DF, and by this we hope to avoid eliminating true eggs that were only detected once. However, since we expect a true egg detection to be more likely to be found in both BF and DF, the object-level OR reduces the overall confidence score of lonely detections. Fig 3C shows examples of the resulting scores for object pairs when object-level combinations are applied.

After calculating the object-level AND/OR scores, the patients are classified as positive or negative on a patient-level, based on the presence of combined bounding boxes at a given confidence score threshold. Subsequently, patient-level sensitivity and specificity are calculated and compared to the TPP targets for each use case, as described above.

### Bootstrapping

We used bootstrapping to gain insights into the variability of our patient-level metrics and to run statistical tests on the results of our models and combinations. To do this, we iteratively resampled the patient population with replacement, re-running our analysis 100 times on random subsets of 40% of our test patients. For each iteration, we found the threshold that resulted in the TPP target specificity, then calculated the patient-level sensitivity using this threshold for the BF-only, DF-only, and combination models. We then used a Kruskal-Wallis test (the non-parametric equivalent of an ANOVA test) with Dunn’s correction for multiple comparisons, to determine whether there were statistically significant differences between the BF and DF models and the combinations. We performed two sets of comparisons: we tested whether the DF model and all the BF-DF combinations were significantly different from the BF model, and we tested whether the BF-DF combinations were significantly different from the DF model alone. All statistical analyses were done using GraphPad Prism (version 10.2.2).

### Comparison with standard light microscopy

We used standard light microscopy results collected in the field during the acquisition of Dataset 2 to evaluate our ML models and our BF and DF combinations. We used the same strategy for patient-level analysis described above but, rather than using the eggs captured and annotated on the images as the ground truth, we considered patients positive if their standard light microscopy counts were above zero, and negative otherwise. We excluded two patients due to a lack of standard light microscopy results for them in our records. We also excluded seven patients that had clear, annotated examples of *Schistosoma* eggs in the images, despite having a standard light microscopy count of zero eggs.

## Results

### ML model performance on Dataset 1 splits

BF and DF models were trained on subsets of Dataset 1. We used 5-fold splits to better assess their performance before training a set of final models for evaluation using Dataset 2. Results for the BF and DF models on these splits are shown in Fig 2, and the results for the BF and DF combinations of those models on these splits are shown in Fig 3, all of these results are at the patient-level.

The average sensitivity at the TPP specificity for TI&S for the 5 splits was higher for DF (84%) than for BF (80%). The targeted sensitivity for this TPP use case is 88%; only one split for BF and two splits for DF reached this requirement. However, for the M&E use case, all of the DF splits and most of the BF splits reached the targeted sensitivity of 75%. For this use case, the average sensitivity was also higher for DF (88%) than for BF (84%).

Using DF alone or BF-DF combinations resulted a 4-10% increase in mean sensitivity at the targeted TPP specificity values. Notably, when applying both object-level and patient-level combinations, all of the splits of the March 2020 dataset met the TPP requirements for sensitivity and specificity for the M&E use case. Despite not reaching the targeted TPP sensitivity for the TI&S use case on all splits of the data, both object- and patient-level combinations increased the average sensitivity, bringing it closer to the WHO targets.

Results for BF and DF models and combinations trained and tested on the 5-fold splits of Dataset 1 using three different architectures (ResNet50, YOLOv5, and YOLOv8) are shown in S2 Fig. For all architectures tested and for both TPP use cases, the DF model and BF-DF combinations had an average performance that was greater than the BF models alone.

### ML model performance on hold-out Dataset 2

**Fig 4:**
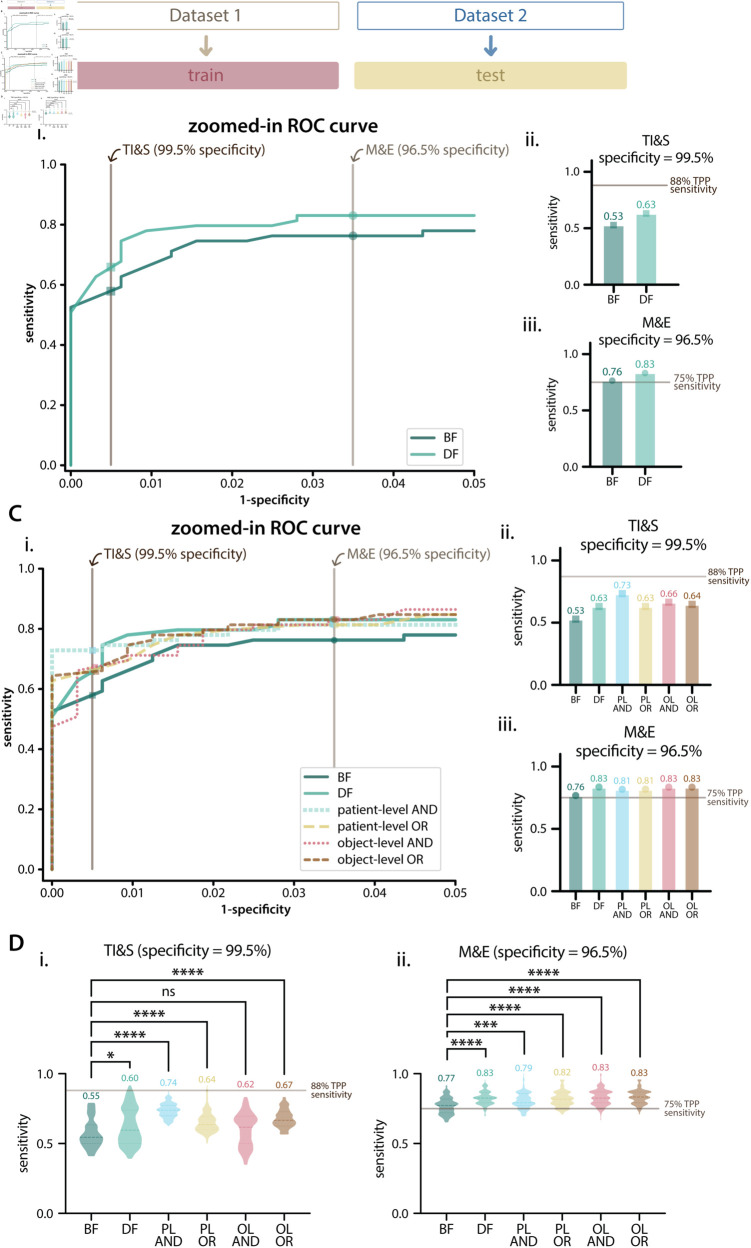
Patient-level results on Dataset 2 as a holdout. **A**: Diagram of data used for training and testing. **B**: Results for brightfield (BF) and darkfield (DF) models trained on Dataset 1 and tested on Dataset 2. Bi: zoomed-in ROC curve showing specificity values from 95% to 100%, with TPP specificity requirements shown as vertical lines. Bii and Biii: patient-level sensitivity for BF and DF models for the TI&S (Bii) and M&E (Biii) use cases, with sensitivity values for each model displayed above each bar and target sensitivity displayed as a horizontal line. **C**: Results for model combinations on Dataset 2. Ci: zoomed-in ROC curve showing specificity values from 95% to 100%, with TPP specificity requirements shown as vertical lines. Cii and Ciii: sensitivity results for BF and DF models and combinations for the TI&S (Cii) and M&E (Ciii) use cases. PL AND is patient-level AND, PL OR is patient-level OR, OL AND is object-level AND, OL OR is object-level OR. **D**: Bootstrapping results on the holdout set for TI&S and M&E TPP use cases. The violin plots show the distribution of patient-level sensitivity values at thresholds resulting in the targeted TPP specificity. Bootstrapping was performed for 100 iterations, with sample size = 40% of the patient population. The dashed lines inside violins show the median of the distribution, dotted lines show the quartiles. The median of each distribution is displayed above each violin. A Kruskal-Wallis test with Dunn’s correction for multiple comparisons was used to compare the BF model with the DF model and the combination models. We report multiplicity-adjusted p-values, “ns” is p > 0.05,   is p ≤ 0.05, ^***^ is p ≤ 0.001, ^****^ is p ≤ 0.0001.

After model training was complete and we confirmed that the performance on Dataset 1 was adequate, we trained models using all of Dataset 1 and tested them on Dataset 2 as a holdout set. This is the scenario that is most realistic and consistent with future diagnostic work in the field, and does not incorporate any information from the test dataset into the training. The patient-level results are shown in Fig 4. A diagram illustrating the data used for training and testing is shown in Fig 4A.

All models and combinations performed worse when trained on Dataset 1 and tested on Dataset 2, compared to the average performance of 5-fold split models trained and tested on subsets of Dataset 1. This is expected, and gives us a better idea of how our trained models would perform with unseen data in future field studies.

Both BF and DF models met the targeted sensitivity for the M&E use case, but they did not meet the targeted sensitivity for the TI&S use case. The DF models performed better than BF when we evaluated the models for both use cases (Fig 4B).

The results when the BF and DF models are combined on an object-level and patient-level are shown in Fig 4C. The full ROC curves and AUC of the BF and DF models and combinations are shown in S3 Fig. All BF-DF combinations reached the target sensitivity for the M&E use case and performed better than the BF model. The patient-level combinations performed slightly worse than DF, and the object-level combinations had the same performance as DF. None of the BF-DF combinations achieved the target sensitivity for the TI&S use case, but all combinations resulted in a sensitivity greater or equal to that achieved with the BF and DF models separately. The greatest increase was achieved when using a patient-level AND combination.

We used bootstrapping to investigate how our patient-level metrics would have varied had the patient population been a subset of what is in Dataset 2 (Fig 4D). The median sensitivity of the bootstrap populations is similar to the sensitivity obtained when testing over the full holdout set. There are statistically significant differences (p ≤0.05) between the BF and DF models, as well as between the BF model and most model combinations (with the exception of object-level AND for TI&S). A statistical comparison between the bootstrapped DF models and combinations is shown in S4 Fig. There was not a significant difference between DF and combination models for the M&E use case, but for the more stringent TI&S use case, some combinations (PL AND and OL OR) were significantly better than DF alone.

Models trained on Dataset 1 and tested on Dataset 2 were evaluated using standard light microscopy results as the ground truth (S5 Fig). The trend of DF models and most BF-DF combinations performing better than BF—seen when using annotated eggs on images as the ground truth—was maintained, with the exception of object-level combinations for the TI&S use case. For the TI&S use case, BF, DF, and patient-level BF-DF combinations performed similarly to when evaluated using the image annotations as the ground truth, but the object-level BF-DF combinations had much worse performance. No model or combination reached the required sensitivity at the TI&S TPP specificity. For the M&E use case, all models and combinations (with the exception of DF) performed slightly worse than when the image annotations were used as the gold standard. All models and combinations reached the required sensitivity at the thresholds that resulted in the required TPP specificity.

### ML model performance on merged dataset (Dataset 3)

**Fig 5:**
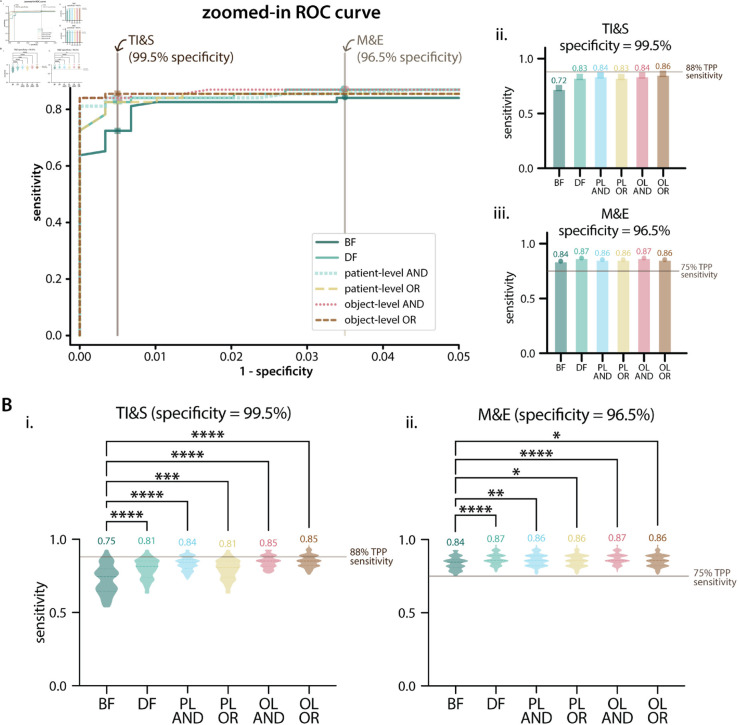
Patient-level results on Dataset 3. **A**: Patient-level results on the test set of Dataset 3. Ai: zoomed-in ROC curve with specificity values ranging from 95% to 100%, with TPP specificity requirements shown as vertical lines. Aii and Aiii: patient-level sensitivity results for BF and DF models and combinations for the TI&S and M&E use cases (at thresholds resulting in TPP specificity). The target sensitivity for each use case is shown as a horizontal line. BF is brightfield, DF is darkfield, PL AND is patient-level AND, PL OR is patient-level OR, OL AND is object-level AND, OL OR is object-level OR. **B**: Bootstrapping results on the test set of Dataset 3 for TI&S and M&E TPP use cases. The violin plots show the distribution of patient-level sensitivity values at thresholds resulting in the targeted TPP specificity. Bootstrapping was performed for 100 iterations, with sample size = 40% of the patient population. The dashed lines inside violins show the median of the distribution, dotted lines show the quartiles. The median of each distribution is displayed above each violin. A Kruskal-Wallis test with Dunn’s correction for multiple comparisons was used to compare the BF model with the DF model and the combination models. We report multiplicity-adjusted p-values, “ns” is p > 0.05,   is p ≤ 0.05, ^***^ is p ≤ 0.001, ^****^ is p ≤ 0.0001.

Overall, the performance of the ML models on Dataset 3 was better than the performance of the models trained on Dataset 1 and tested on Dataset 2 (Fig 5). When evaluated at thresholds that met the TPP target specificity, all contrasts and combinations met the TPP target sensitivity for the M&E use case. For the TI&S use case, no contrast or combination met the target TPP sensitivity. The object-level OR combination was the closest, with only 2% lower sensitivity than the target.

We performed bootstrapping to gain insight on the variability of the patient-level metrics. For the TI&S use case, DF and all of the contrast combinations performed significantly better than BF. The object-level combinations (AND and OR) had both the highest median sensitivity and the tightest distributions. Notably, the third quartile for both of these distributions was above the sensitivity targeted by the TPP (88% sensitivity). 29/100 iterations for object-level AND and 30/100 iterations for object-level OR had a sensitivity above the TPP target for TI&S.

Fig 5Bii shows the sensitivity distributions for the M&E use case. DF and all of the combinations performed significantly better than BF. Notably, for all of the contrasts and combinations and for all of the 100 iterations, the models had a sensitivity above or equal to that targeted by the TPP (75% sensitivity) at a threshold that resulted in the targeted specificity.

A comparison between the bootstrapped DF models and combinations is shown in S4 Fig. There was not a significant difference between DF and combination models for the M&E use case, but for the more stringent TI&S use case, most combinations (with the exception of patient-level OR) were significantly better than DF alone.

## Discussion

Diagnostic technologies that are low-cost, simple to use, and achieve WHO performance metrics are needed to advance schistosomiasis control and elimination goals. The development of mobile phone-based microscopes for image-based diagnosis of *S. haematobium*, such as the SchistoScope, partially achieve those goals through their portability and simplicity. However, the best strategy for automated egg detection and patient diagnosis for mobile microscopes with moderate resolution has been unclear, as many existing ML models rely on images collected with high-resolution imaging systems. Moderate resolution systems, including the SchistoScope, may need additional information to achieve the combination of sensitivity and specificity needed for field applications. This paper highlights the potential of DF as a means to break the zero-sum trade-off between accuracy and practicality, by enabling portable, lower resolution systems, to support high accuracy detection.

We use multi-contrast images of patient urine samples containing *S. haematobium* acquired in endemic regions of Côte d’Ivoire using the SchistoScope to train ML models for automated diagnosis. Importantly, the ML models we trained can be efficiently deployed on mobile devices. We find that DF models alone and combinations of BF and DF models lead to greater performance than BF alone, which is the typical contrast used to identify eggs with light microscopy. The combinations of BF and DF models meet the WHO target sensitivity and specificity for monitoring and evaluation of schistosomiasis control programmes, with DF consistently showing better performance than BF. A relatively small dataset of less than 1000 images was sufficient to train the models and demonstrate improved diagnostic performance, taking advantage of the availability of pre-trained, off-the-shelf ML models that can be used for fine-tuning to a particular application. We achieved performance improvements by using DF and BF-DF combinations to train models of three different ML architectures: YOLOv8, YOLOv5, and ResNet50. We used YOLOv8 most heavily in this work, but other algorithms could be more suitable depending on the specific application. YOLOv5 models could be more appropriate for resource-constrained applications or for running on devices with limited computational power, while YOLOv8 models would be suitable for high-accuracy applications [35]. While the best ML architecture for detection of *Schistosoma* eggs or for multi-contrast ML is not yet clear, we expect our findings to generalize to other commonly used ML architectures and models based on the consistent performance improvements seen with DF on the three architectures tested.

Our multi-contrast machine learning approach benefited from BF and DF images providing complimentary information about the *Schistosoma* eggs, with brightfield contrast reporting light absorption by the sample and darkfield contrast showing scattering by sample edges and other features. The use of darkfield imaging for ML-based disease identification and image classification has shown promise in other fields [36–47]. However, to our knowledge, this is the first demonstration of its combined use with brightfield as a means to improve diagnostic performance in the context of limited data for diagnosis of neglected diseases. Darkfield, or pseudo-darkfield, can be easily (and fairly inexpensively) implemented in a standard light microscope by adding an oblique or annular illumination source, or by blocking illumination angles that are captured by the imaging lens, an example of which is shown in [48]. Hence, DF imaging could be implemented by other groups integrating ML with portable microscopy for diagnosis of *S. haematobium* and other diseases with egg-based diagnostics, such as *Schistosoma mansoni* and soil-transmitted helminths. In fact, DF imaging alone can be helpful for semi-automatic diagnostic strategies where clinicians or field technicians make diagnostic calls based on digitized images of patient samples. Our annotators and clinical collaborators noted that they preferred annotating/evaluating DF images because *S. haematobium* eggs are easier to identify in DF versus the traditional BF contrast.

In cases where a microscopy system supports both BF and DF imaging, these can be combined in relatively simple ways to get better ML results. We showed that simple, boolean combinations of models trained on images of different contrasts can lead to improvements in performance. Our bootstrapping results suggest that combining BF and DF models can result in more stability, especially in more stringent diagnostic use cases. This is particularly evident in the tighter spread over bootstraps for most BF-DF combination models in the TI&S use cases (Figs 4Di and 5Di) This was also evident when performing statistical comparisons between the DF models and the combinations (S4 Fig), where some combinations performed significantly better than DF for the TI&S use case. Incorporating additional contrasts, such as differential phase contrast and fluorescence, into portable and low-cost microscopes could provide additional sample information that might further improve multi-contrast machine learning performance. In particular, the autofluorescence of *Schistosoma* eggs and other parasites makes this an attractive direction for future device development. Improvements in ML model development could also advance the goal of high performance detection with lower resolution images, including altering the model architecture to train on images of both contrasts simultaneously, increasing hyperparameter optimization, and training on more egg images. As the field of ML continues to evolve rapidly, novel models and architectures could also lead to performance improvements, potentially reaching the TI&S target metrics.

An additional strategy for multi-contrast ML could be an analytical combination of BF and DF images (i.e., merging BF and DF into a single input image), followed by model training. Given our use of transfer learning with models that use 3-channel RGB images, we attempted analytical combination by converting BF and DF images to greyscale and using these to create 3-channel images (e.g., [BF(greyscale), BF(greyscale), DF(greyscale)]), followed by model training with these new images. These models did not perform better than our BF models, we suspect due to differences in focus and slight movements between BF and DF image acquisition. Analytical combination of BF and DF could be attempted on a dataset where focus was kept constant during image acquisition, or by using bespoke ML models with a 6-channel input.

Our models trained on Dataset 1 and tested on Dataset 2 had similar performance when using the annotated images and the standard light microscopy results as the ground truth. We observed some discrepancies between the image annotations and light microscopy counts, where some patients were classified as positive via microscopy and negative via egg annotations on the images, and vice versa. Discrepancies could arise from inhomogeneous egg distribution in urine samples, especially in low-parasitemia patients. Cases where patients were positive via light microscopy but had no annotated eggs on their images could be due to imperfect capillary capture efficiency [22] or due to focus errors or dirty capillaries during image acquisition. Patients that were negative via standard light microscopy but had annotated eggs in the SchistoScope images could be due to field technicians missing eggs during sample examination, given that each sample was only evaluated by one technician. However, ML models and combinations reached the required TPP sensitivity and specificity when evaluated for the M&E use case, with DF models and BF-DF combinations performing better than BF models. These results show the potential of this strategy for future field studies, and highlight the usefulness of incorporating DF imaging in schistosomiasis diagnostics.

An important next step to validate the usefulness of multi-contrast machine learning will be to do live field testing of ML models loaded onto the SchistoScope or its successor. This will require exporting our ML models to a mobile phone-compatible format to evaluate performance and processing time. Any future field deployment will also require selecting confidence score thresholds in advance and providing patient-level diagnosis based on them.

We observed ML model performance improvements when training on the combined dataset (Dataset 3) compared to training on data from one site (Dataset 1) and testing on data from the second site (Dataset 2), which is what we would expect if there was a shift in distribution between Dataset 1 and Dataset 2 due to their acquisition during different field visits. By using Dataset 3, we saw how our models would perform in the ostensibly best available case, where maximum training data is used and testing is done in-distribution. Variations in performance between study sites have been observed during field-testing of other diagnostic products, including those that use more standardized sample processing techniques (e.g., thin smear for malaria) [31]. Future field deployments of the SchistoScope could benefit from real-time updates of the ML models to accommodate inter-clinic variability and uneven algorithm accuracy at new sites due to distribution shifts in training and testing populations. It is also worth noting that the WHO TPP for TI&S is in the context of disease elimination, which generally implies lower parasitemia distributions, making it harder to hit the sensitivity targets [28]. With further improvements to the model, we expect our multi-contrast strategy to be particularly promising in this context.

## Conclusion

Mobile phone-based microscopy platforms in conjunction with multi-contrast machine learning and novel sample preparation techniques can be used for rapid, sensitive, and portable diagnosis of *S. haematobium* that meets WHO diagnostic requirements. Performance of ML models to identify *Schistosoma* eggs can be significantly improved by adding DF imaging to standard BF microscopes, which requires minimal changes in microscope optics and no additional sample preparation. Multi-contrast machine learning offers a practical means to improve performance of low-cost, automated diagnostics for *S. haematobium* egg detection and could be applied to other microscopy-based diagnostics.

## Supporting information

S1 Fig Precision-recall curves for all splits of Dataset 1. Precision-recall curves for the BF and DF models tested on the 5-splits of Dataset 1.
(PDF)

S2 Fig ResNet50, YOLOv5, and YOLOv8 results on splits of Dataset 1. Results on the 5-splits of Dataset 1 for ML models trained using three different architectures.
(PDF)

S3 Fig ROC curve and AUC for Dataset 2. Receiver operator characteristic curves (ROC) and area under the curve (AUC) for Dataset 2 ML models and combinations.
(PDF)

S4 Fig Statistical comparison between DF models and combinations. Statistical comparison between DF models and BF-DF combinations for Dataset 2 and Dataset 3 bootstrapping results.
(PDF)

S5 Fig ML model results compared to standard light microscopy as the ground truth. Patient-level results of ML models tested on Dataset 2 and using standard light microscopy counts as the ground truth.
(PDF)

S1 Table Dataset 1 image information. Table with information about Dataset 1 images, including image name, patient number, contrast, and number of annotated eggs on an image-level and patient-level.
(CSV)

S2 Table Dataset 2 image information. Table with information about Dataset 2 images, including image name, patient number, contrast, and number of annotated eggs on an image-level and patient-level.
(CSV)

S3 Table Dataset 1 S. *haematobium* egg annotations. Table with bounding box xy coordinates for *S. haematobium* eggs and “doubtful” objects annotated on the images of Dataset 1.
(CSV)

S4 Table Dataset 2 *S. haematobium* egg annotations. Table with bounding box xy coordinates for *S. haematobium* eggs and “doubtful” objects annotated on the images of Dataset 2.
(CSV)
