# Supplementary material for: Multi-contrast machine learning improves schistosomiasis diagnostic performance
Source: PLoS Negl Trop Dis. 2025 Aug 4;19(8):e0012879. doi: 10.1371/journal.pntd.0012879 (PMC12334053; doi:10.1371/journal.pntd.0012879)
Supplement: S1 Fig — Precision-recall curves for the BF and DF models tested on the 5-splits of Dataset 1. (PDF) [file pntd.0012879.s001.pdf]

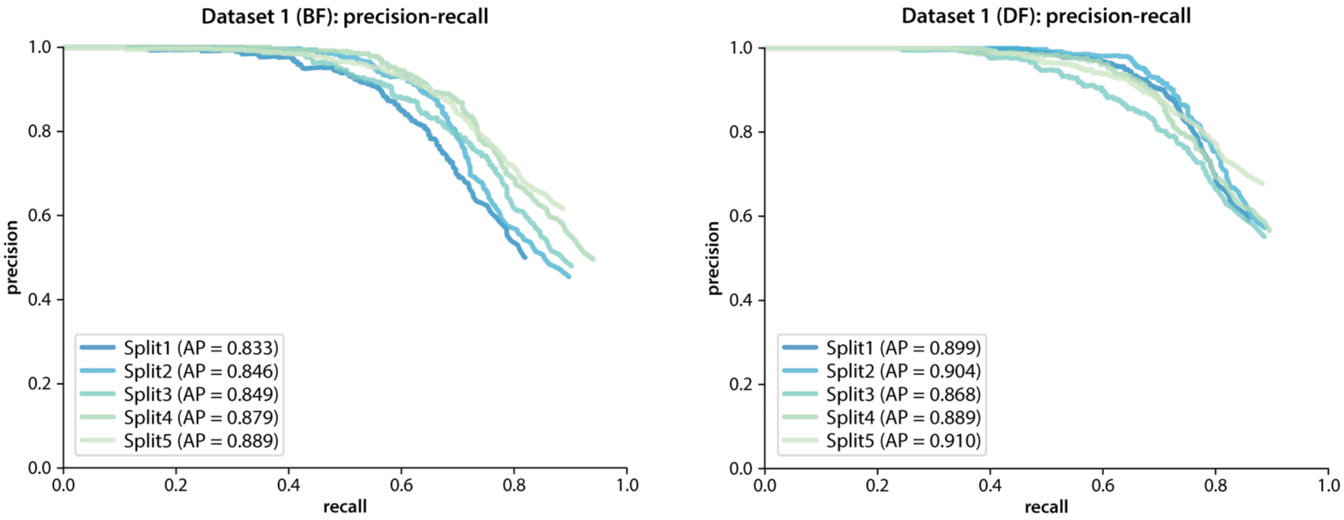

S1 Fig. Precision-recall curves for all splits of Dataset 1

Left: precision-recall curves for the test set of all splits of Dataset 1 using the brightfield ML model. The inset on the bottom left shows the average precision (AP) for each split. Right: precision-recall curves for the test set of all splits of Dataset 1 using the darkfield ML model. The inset on the bottom left shows the average precision (AP) for each split.
