# Supplementary material for: Multi-contrast machine learning improves schistosomiasis diagnostic performance
Source: PLoS Negl Trop Dis. 2025 Aug 4;19(8):e0012879. doi: 10.1371/journal.pntd.0012879 (PMC12334053; doi:10.1371/journal.pntd.0012879)
Supplement: S2 Fig — Results on the 5-splits of Dataset 1 for ML models trained using three different architectures. (PDF) [file pntd.0012879.s002.pdf]

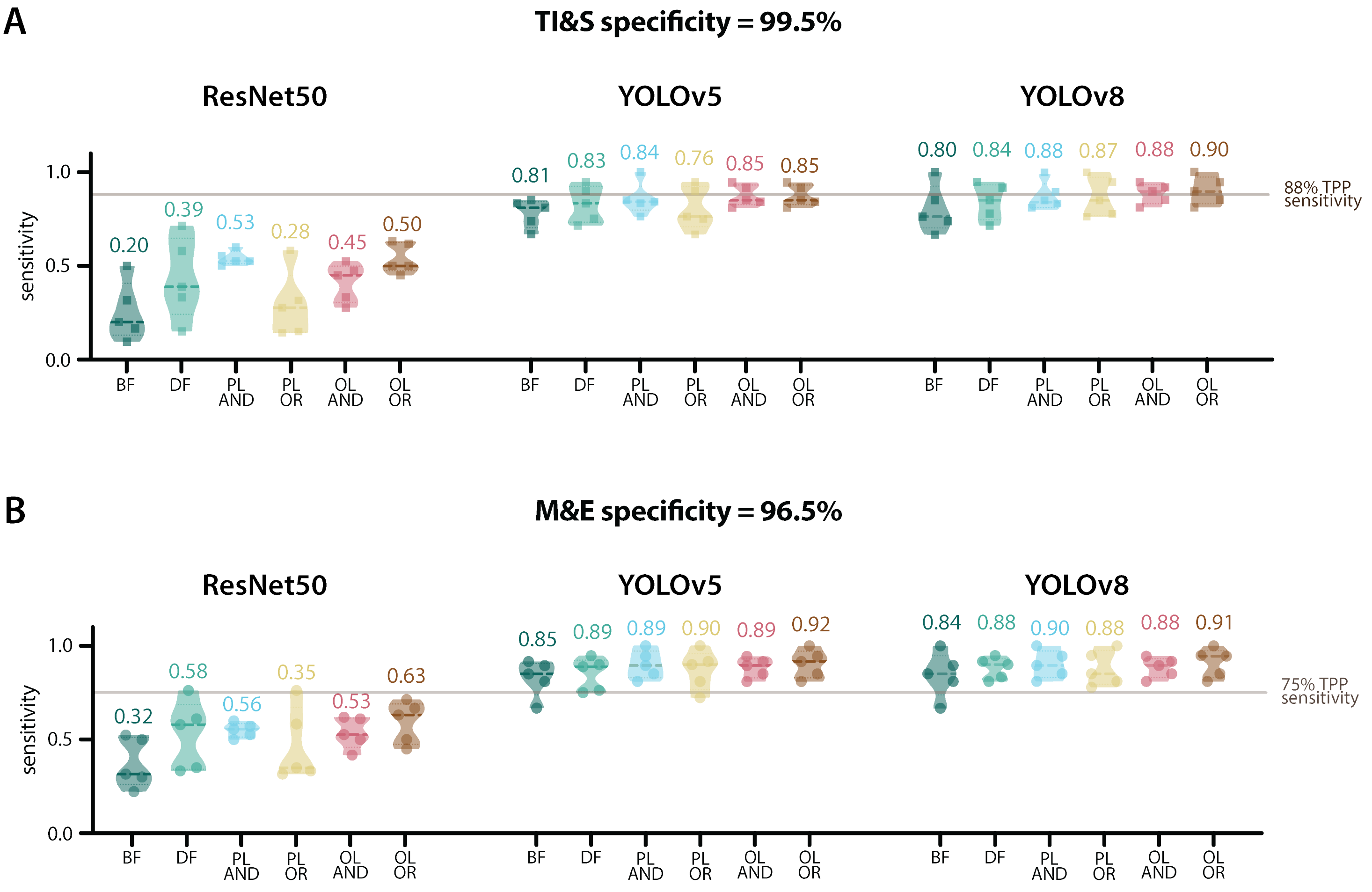

S2 Fig. ResNet50, YOLOv5, and YOLOv8 results on splits of Dataset 1
Violin plots showing the patient-level sensitivity values for the 5 splits of Dataset 1 for the TI&S (**A**) and M&E (**B**) TPP use cases. Results are shown for models trained using three different architectures: ResNet50 (left), YOLOv5 (middle), and YOLOv8 (right, results also shown in Fig 3D and E). BF is brightfield, DF is darkfield, PL AND is patient-level AND, PL OR is patient-level OR, OL AND is object-level AND, OL OR is object-level OR.
