# Supplementary material for: Multi-contrast machine learning improves schistosomiasis diagnostic performance
Source: PLoS Negl Trop Dis. 2025 Aug 4;19(8):e0012879. doi: 10.1371/journal.pntd.0012879 (PMC12334053; doi:10.1371/journal.pntd.0012879)
Supplement: S3 Fig — Receiver operator characteristic curves (ROC) and area under the curve (AUC) for Dataset 2 ML models and combinations. (PDF) [file pntd.0012879.s003.pdf]

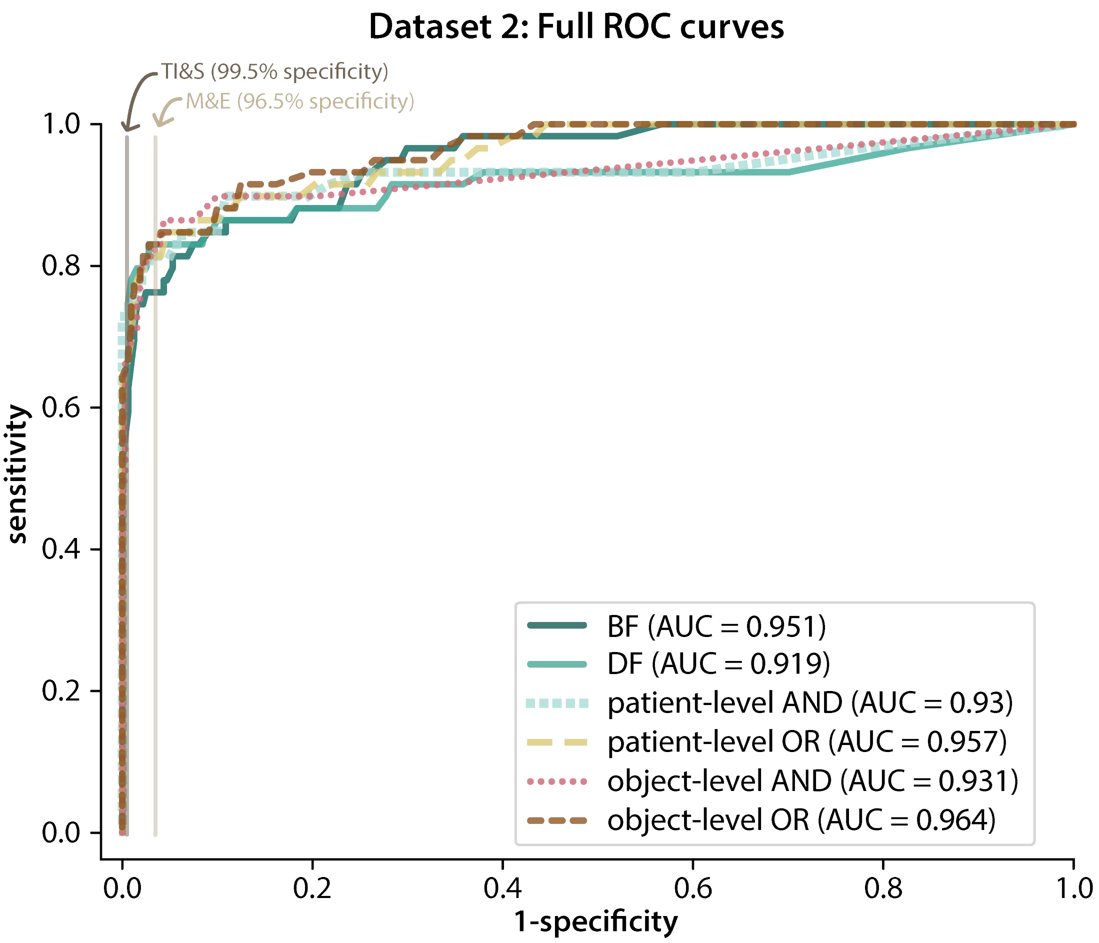

S3 Fig. ROC curve and AUC for Dataset 2

Receiver operator characteristic curves (ROC) and area under the curve (AUC) for Dataset 2 models and combinations. The AUC for each model and combination is shown in the inset on the bottom right. The required specificity values for the TI&S and M&E TPP use cases are displayed as vertical lines.
