# Supplementary material for: Multi-contrast machine learning improves schistosomiasis diagnostic performance
Source: PLoS Negl Trop Dis. 2025 Aug 4;19(8):e0012879. doi: 10.1371/journal.pntd.0012879 (PMC12334053; doi:10.1371/journal.pntd.0012879)
Supplement: S5 Fig — Patient-level results of ML models tested on Dataset 2 and using standard light microscopy counts as the ground truth. (PDF) [file pntd.0012879.s005.pdf]

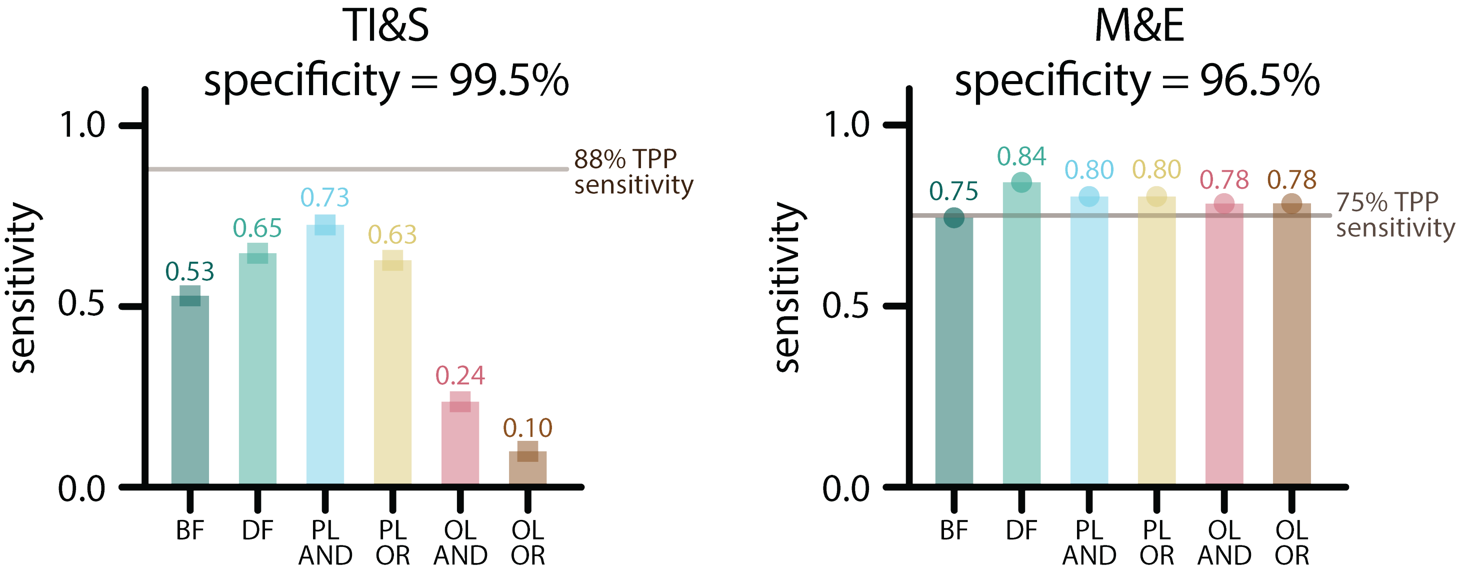

S5 Fig. ML model results compared to standard light microscopy as the ground truth
Patient-level results of ML models tested on Dataset 2 and using standard light microscopy results as the ground truth. Plots show the patient-level sensitivity when using confidence score thresholds that resulted in the TPP specificity for the TI&S (left, 99.5% specificity) and M&E (right, 96.5% specificity) use cases. Sensitivity values for each model are displayed above each bar, and TPP target sensitivity for each use case is displayed as a horizontal line. Nine patients from Dataset 2 were excluded from this analysis: two patients for whom a light microscopy count was not available, and seven patients who had a negative light microscopy result but had clear *S. haematobium* eggs shown in the patient images. BF is brightfield, DF is darkfield, PL AND is patient-level AND, PL OR is patient-level OR, OL AND is object-level AND, OL OR is object-level OR.
