# Supplementary material for: Multi-contrast machine learning improves schistosomiasis diagnostic performance
Source: PLoS Negl Trop Dis. 2025 Aug 4;19(8):e0012879. doi: 10.1371/journal.pntd.0012879 (PMC12334053; doi:10.1371/journal.pntd.0012879)
Supplement: S4 Fig — Statistical comparison between DF models and BF-DF combinations for Dataset 2 and Dataset 3 bootstrapping results. (PDF) [file pntd.0012879.s004.pdf]

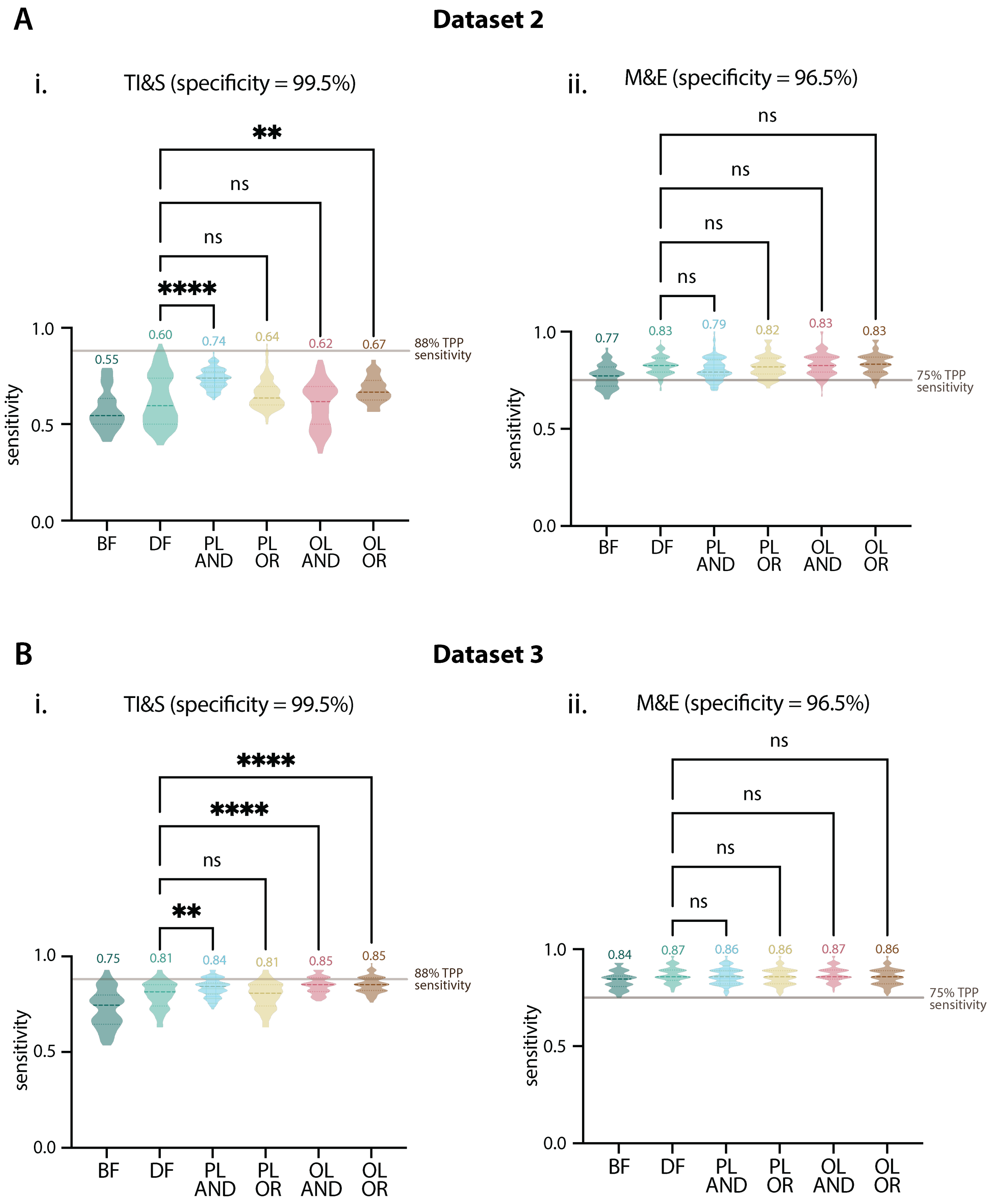

S4 Fig. Statistical comparison between DF models and combinations
Bootstrapping results for Dataset 2 and Dataset 3, showing results of Kruskal-Wallis test with Dunn's correction comparing the DF model with the combination models.
A: Bootstrapping results for Dataset 2 (also shown in Fig 4D) and statistical tests for TI&S (i.) and M&E (ii.) **B**: Boostrapping results for Dataset 3 (also shown in Fig 5B) and statistical tests for TI&S (i.) and M&E (ii.) We report multiplicity-adjusted p-values, “ns” is p>0.05, * is p≤0.05, *** is p≤0.001, **** is p≤0.0001.
